# Parental Anxiety Disorders and Their Impact on Dental Treatment in Children Aged 4 to 13 Years: A Cross-Sectional Observational Study

**DOI:** 10.3390/jcm14061869

**Published:** 2025-03-10

**Authors:** Gloria Bayón, Fabiola Stiernhufvud, David Ribas-Pérez, María Biedma Perea, Asunción Mendoza Mendoza

**Affiliations:** Department of Stomatology, Faculty of Dentistry, University of Seville, Avicena Street s/n, 41009 Seville, Spainmbiedma1@us.es (M.B.P.); amendoza@us.es (A.M.M.)

**Keywords:** dental anxiety, dental fear, caries index, dental treatment

## Abstract

**Introduction:** Children with dental fear and/or anxiety will use all available means to avoid or delay dental treatment, which can cause a deterioration in their oral health. A close relationship has been demonstrated between parents’ fear and/or anxiety about the dentist and the development of dental anxiety in children. **Objective:** Our aim is to evaluate the anxiety of children’s parents and the factors that influence the prediction of anxiety and children’s behavior, as well as its impact on the risk of caries. **Method:** This is a descriptive cross-sectional study. For data collection, scientifically validated questionnaires were used for parents (*n* = 101) and children (*n* = 101). Statistical analysis was performed using the Chi2 test, the independent sample t test, and the Mann–Whitney test. **Results:** A direct relationship (*p* = 0.095) was found between the Corah test and the Venham test, as well as statistical significance (*p* = 0.035) between the STAI-Trait and the Venham test. The type of treatment the child is going to undergo is a determining factor in parental anxiety. A total of 85% of the patients exhibited positive behavior regardless of the degree of parental anxiety. **Conclusions:** The relationship between the anxiety of the parent and the child was very limited and restricted to specific cases; direct associations were found between the oral state of the child and the anxiety of the parents.

## 1. Introduction

Fear and anxiety are correlated emotional states. Fear is the reaction generated by a real or imminent danger and is linked to the stimulus that produces it; therefore, it is an objective reaction. On the other hand, anxiety is the anticipatory state of a future danger that has not yet occurred and that we do not know will occur; it is considered a “subjective fear”, as there is no apparent cause that generates it [1,2]. These emotional processes are, to a certain extent, essential to the human affective repertoire because they enhance performance in motor, physiological, and cognitive tasks. In this context, they will be considered adaptive phenomena. However, when these emotions exceed levels that are considered “normal” and compromise an individual’s performance of daily activities, they will be considered pathologies [3].

Dental anxiety is defined as a feeling of apprehension about dental treatment [4]. It is an excessive, negative, and unreasonable emotional state experienced by patients before, during, or after dental treatment [5]. In contrast, dental fear refers to an unpleasant emotional reaction to specific threatening stimuli that occur in situations associated with dental treatment [5].

Childhood behavior associated with dental anxiety and fear has been one of the great challenges in pediatric dentistry. The emotional state of the child during dental treatment can create difficulties in terms of its evaluation due to, among other things, the child’s immaturity in communicating his feelings [6].

Currently, there is a high percentage of generalized dental anxiety in the population. It is estimated that 264 million people suffer from anxiety, a prevalence that has increased by up to 15% from 2005 to 2015 as a result of population growth and an increase in life expectancy [7]; it is recognized by the World Health Organization (WHO) as a true pathology [8,9,10]. In Spain, according to the latest report (December 2020) from the primary care clinical database (BDCAP) [11], anxiety is the most frequent mental health problem in the Spanish population, affecting 88.4% of women and 45.2% of men aged between 35 and 84 years.

There are multiple factors that cause or trigger dental anxiety, namely, the age and sex of the patient [5,12,13], the dental treatment to which he or she will be subjected [14], the teaching style and family structure [15], the number of siblings and their birth order [16], and the anxiety of the parents or progenitors. Therefore, it is important to inform and guide parents about the oral health of their children and the influence of their feelings on those of the child. Less anxious children tend to accept more easily the procedures to which they will be subjected, which allows for the success of the dental treatment [17].

It has been shown that by carrying out tests on dental anxiety prior to treatment, it is possible to identify patients who suffer from this disorder, thereby allowing for greater cooperation and a reduction in the anxiety levels of these patients. However, very few professionals carry out this type of testing before dental treatment, since most rely on their experience and intuition to assess a patient’s dental anxiety level. In contrast, pediatric dentists have been shown to be more successful in identifying anxious and non-anxious pediatric patients, leading to better outcomes when performing these types of treatments on children [18,19,20].

In the current literature, there are different scales to assess the anxiety of guardians or parents, namely, the Corah Dental Anxiety Scale [21,22] and its variant, the Modified Corah Dental Anxiety Scale (MDAS) [23], the Early Childhood Oral Health Impact Scale (ECOHIS) [24], and/or the Dental Anxiety Inventory (IDATE) [25].

On the other hand, dental anxiety in children can be assessed using the Children’s Fear Survey Schedule (CFSS) [26], designed by Scherer and Nakamura to study the distribution and etiology of dental fear in children, and/or the Venham Clinical Anxiety Rating Scale [27,28], which consists of a series of cartoon figures representing various emotional states. This approach allows the child to identify with each of the situations shown, resulting in a simple yet valid and reliable scale of the child’s response to situational stress.

The aim of the present study is to establish the possibility of a relationship between the emotional state of the parents and the child’s behavior during dental practice, as well as the risk of caries that the child presents.

## 2. Materials and Methods

### 2.1. Design and Sample of This Study

A descriptive cross-sectional study was carried out with 202 patients, including 101 guardians or parents and 101 children aged between 4 and 13 years, who attended the pediatric dentistry services of a private clinic located in the south of Badajoz (Spain). This age range is justified since a minimum level of knowledge is necessary on the part of the child to be able to answer the questions in the questionnaire.

In the statistical analysis, the proposed sample will be adjusted, and a 95% confidence interval will be proposed with a 5% margin of error; the data may be modified according to the circumstances for a better adaptation of the work.

### 2.2. Data Collection

Following approval by the ethics committee of the Andalusian Government, inclusion and exclusion criteria will be established, and data collection will be out; questionnaires and anxiety scales that have already been scientifically researched and previously translated into Spanish, with high reliability, easy application, and low cost will be used. Data collection will be carried out through interviews with parents or guardians, as well as interviews and clinical–radiographic examinations of children. These will be part of the dental procedure that the patient will undergo and will also serve to determine the caries index (dft/DMFT, as required) of each patient, thus helping us understand its influence on the level of anxiety.

Reading the information sheet and signing the informed consent by parents or legal guardians was essential for participation in this research study.

### 2.3. Dental Anxiety in Children

The scales used to verify dental anxiety in children are as follows:

➢ Venham Clinical Anxiety Rating Scale [27,28]: Performed on children between the ages of 4 and 8 years, inclusive. From each illustration, the patient must choose one of the figures, which will determine their emotional state.
➢ Child and Adolescent Fear Scale (CFSS) [26]: Designed by Scherer and Nakamura to study the distribution and etiology of dental fear in children. This test will be performed on patients over 8 years of age.
➢ Frankl Behavior Scale [29]: In 1962, Frankl devised a behavior scale to study children’s reactions to being separated from their parents in the dental office. For this reason, Frankl analyzed children’s behavior in each of the circumstances involved in a first visit (clinical examination, X-ray, prophylaxis, etc.) and a treatment visit (anesthesia injection, cavity preparation, filling, etc.), classifying the patients according to their behavior as definitely positive, slightly positive, slightly negative, or definitely negative.
➢ Collection of vital signs: The pulse will be recorded, with the help of a Homiee Pulse Oximeter^®^, and, using the North Carolina Behavior Scale [30], parameters will be analyzed, including the movements of the legs and arms, the presence of crying, and any oral and/or physical resistance from the child.

### 2.4. Dental Anxiety in Guardians or Parents

The scales used to verify dental anxiety in guardians or parents are as follows:

➢ Modified Corah Dental Anxiety Scale (MDAS) [23]: There are several methods for measuring dental anxiety, one of them being the Modified Corah Dental Anxiety Scale [23], which was later expanded and modified. Each question presents five alternative responses evaluated on a scale from 1 to 5, indicating the absence of anxiety and the highest level of anxiety, respectively. The score ranges from 5 (no anxiety) to 25 (high anxiety).
➢ The Early Childhood Oral Health Impact Scale (ECOHIS) [24]: In order to verify the mother’s perception in relation to her children, we used the ECOHIS, or the Early Childhood Oral Health Impact Scale [24]. According to the WHO, this scale includes a section on child impact (domains of symptoms, function, psychology, and self-image/social interaction) and a section on family impact (domains of distress and family function). The questionnaire contains 13 questions, whose answers include (a) never, (b) almost never, (c) occasionally, (d) often, (e) very often, and (f) I do not know. In addition, 2 extra questions were added to the questionnaire so that parents could rate their children’s general and oral health.
➢ Inventory of Anxiety (IDATE) [25]: It consists of two scales obtained from a questionnaire designed to measure two different anxiety concepts: state anxiety (state A) and trait anxiety (trait A). The trait anxiety scale consists of 20 statements that require subjects to describe how they feel in general. The state anxiety scale also consists of 20 statements from individuals to indicate how they feel at a specific time. For each statement, the subject must select one of the four alternatives to indicate how he or she feels: not at all; a little; enough; and a lot (on the state A scale); or almost never; sometimes; frequently; and almost always (on the trait A scale). These questionnaires were re-adapted by the examiner, eliminating questions that were not considered appropriate for this study and limiting the questionnaire to 10 general statements (state A) and 10 specific ones (trait A), while also adding several questions during the treatment visits.

MDAS [23] and the IDATE [25] will be filled out on 3 occasions by the parents (first visit and first and last day of treatment), while the ECOHIS questionnaire [24] and the fear test [26], given to children over 8 years old, will only be filled out during the first visit, as they consist of more generalized questions.

The level of anxiety of the parents or guardians in relation to the type of treatment that the child will undergo will also be analyzed in order to assess whether the parents’ anxiety varies depending on the type of treatment to be carried out.

### 2.5. Caries Analysis

The caries examination was carried out by a single calibrated operator through visual and radiographic examination, which was subsequently recorded in an odontogram. The calibration was carried out according to the guidelines described by the WHO [31], where the indices used to analyze dental caries consider the tooth as a unit. Thus, patients who have only temporary dentition will be assigned the cod index, while those who have permanent dentition will be assigned the DMFT index. Therefore, those who have mixed dentition will be assigned the cod and DMFT indices independently, depending on the pieces present.

The risk of caries will be determined according to the table of caries severity indicators provided by the WHO [32].

## 3. Results

**Figure 1:**
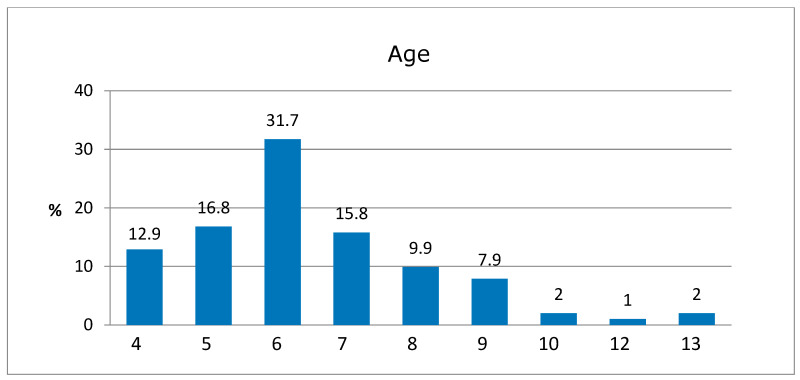
Age distribution of the sample.

The sample consisted of 101 adults (parents or guardians) and 101 children between 4 and 13 years of age who were eligible for dental treatment; the mean age of the study population was 6.4 years. Of the total sample, 46.5% were boys and 53.5% were girls, with the following age distribution (Figure 1):

According to their age, most of the patients had primary dentition (DT) (45.5%) or mixed dentition (DM) (53.5%), and only one had permanent dentition (DP). For the 100 children with DT or DM, where the evaluation of the cod index was possible, a mean of 6.7 ± 2.7 was obtained. For the 55 children with DM or DP, the mean DMFT was 0.4 ± 0.9, as shown in Table 1.

The risk of caries in children with DT is very high in 76.1% of cases.

In the analysis of parental anxiety, 67.3% of parents are classified as having “low” anxiety, and 31.7% are classified as having “moderate” anxiety. Only one parent presents “high” anxiety, according to the ECOHIS. As in the Corah and IDATE tests, neither parent exhibits a pathological state of anxiety.

In the analysis of anxiety and behavior in children, the fear test (CFSS > 8 years) reveals that 30.8% of patients are classified as having “no fear” and 69.2% as having “little fear”. Similarly, in the Venham test (<8 years), 96.6% are classified as “non-anxious” children.

Regarding children’s behavior, according to the Frankl scale, most of the children exhibited very positive behavior during the interventions (first visit to the dentist, T1; first treatment visit, T2; and second treatment visit, T3) (Table 2).

As for crying and limb movement, an increase was found as the treatment progressed (Table 3).

**Figure 2:**
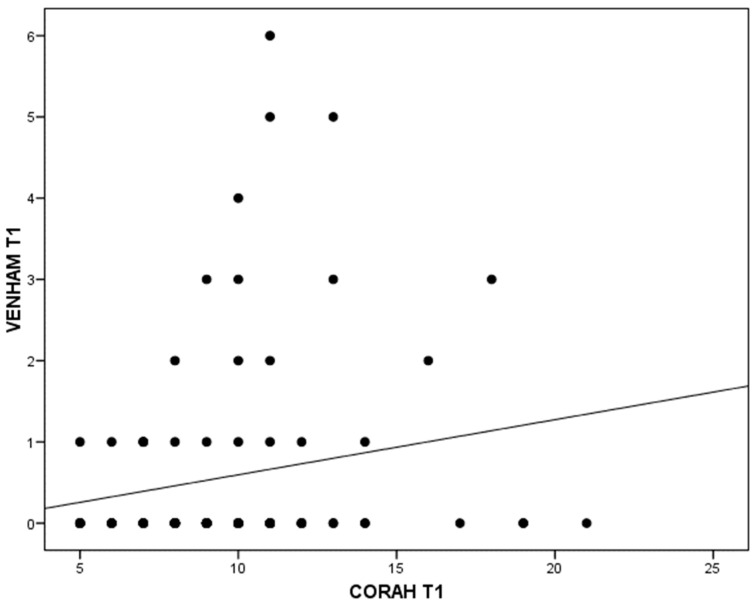
Relationship between parental Corah test and children Venham test at the first visit.

When relating the different tests carried out on the guardians to the tests and data collected from the children, only a weak correlation (r = 0.18; *p* = 0.0095) was detected between the anxiety of the guardians (Corah) and that of the children (Venham < 8 years) at the first visit (Figure 2).

**Figure 3:**
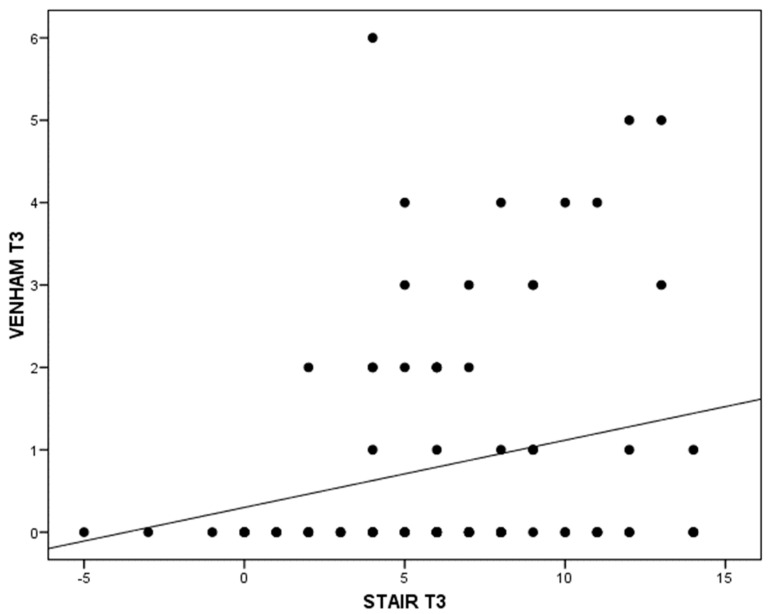
Relationship between parental STAI-trait test test and children Venham test at the third visit.

As with the correlation between Venham and STAI-Trait at the third visit, where statistical significance is reached (*p* = 0.035), it can only be assessed as “weak” (r = 0.22). The higher the parents’ trait anxiety score, the higher the children’s Venham score at the last treatment visit (Figure 3).

The type of treatment is significantly associated with a higher level of anxiety among the parents, as shown in the following table (Table 4):

According to ECOHIS, 83.3% of parents reported “moderate-high anxiety” if a tooth extraction was performed, compared to only 29.5% otherwise. Pulpectomy also increased the rate of anxiety in three of the parents interviewed (Table 5).

According to Corah, 90% of parents experienced “moderate or greater anxiety” when the treatment to be performed was a pulpotomy. In another case, only 50.6% experienced this level of anxiety. Regarding the STAI-State/STAI-Trait, significant relationships were found at the second treatment visit, when the treatment to be performed was a filling or a pulpectomy, as shown in the following table (Table 6):

Regarding the risk of caries, although statistical significance is not reached, a weak correlation is suggested between the cod index and the ECOHIS score; in a direct sense, the higher the ECOHIS, the higher the caries rate (Table 7).

**Figure 4:**
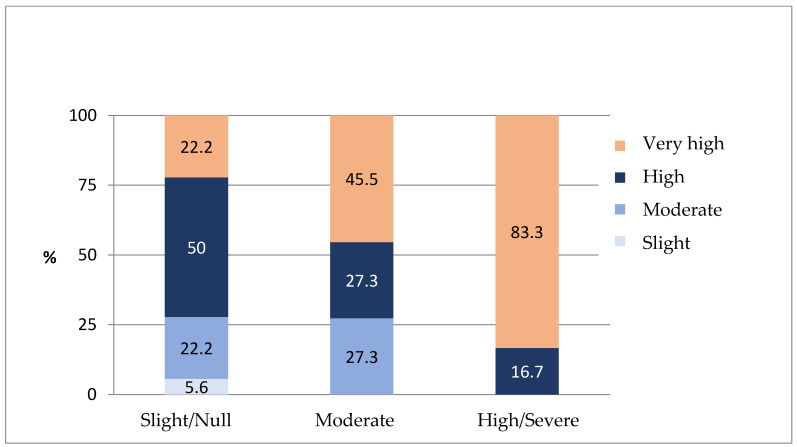
Caries risk in DT according to CORAH Parents Anxiety Scale.

The risk of caries in DT (cod), is significantly correlated with the Corah score of the parent, as shown in the following figure (Figure 4):

Regarding the risk of caries vs. STAI-State, no relevant correlation was found. However, regarding the STAI-Trait, a higher risk of caries was found in DM if the parents scored higher on the STAI-Trait (Table 8).

## 4. Discussion

In our study, we have shown that guardians or parents with fear or anxiety about the dentist can transmit these unconstructive emotions to their children. This can affect the child’s behavior during visits to the dentist (especially during the first visit), but not the child’s development of anxiety or fear symptoms; a weak relationship was observed between the child’s anxiety and that of the parents when we compared the different tests conducted during each visit to the dentist. However, given the small sample size, we cannot reject the null hypothesis.

Petróvic et al. (2024) [33] also show, in their study, that the emotional state of the parents will influence the child’s behavior, either positively or negatively; however, this will not result in less or greater anxiety for the dentist, at least not in all cases. On the contrary, the studies by Rames et al. (2024) [34] and Besiroglu et al. (2024) [35] show that children’s nervousness during dental treatment is mainly caused by the mother’s anxiety. Greater fear and anxiety are found in young children than in adolescents, despite the fact that the latter may have been exposed to more traumatic dental episodes previously [17,36].

These results also coincide with our study, in which the majority of patients who underwent the CFSS were not afraid of the dentist. In contrast, despite the data obtained from this test, the question that generated the greatest fear in patients (>8 years old) was the one related to “needles and injections”. This coincides with the Corah scale administered to parents, where the question with the highest rating was the one related to the “application of an injection” and/or “use of a turbine”, as well as the results obtained in other recent studies [37,38].

Numerous articles demonstrate higher levels of anxiety in girls than in boys [17,39,40]. However, in our study, we did not find differences in terms of sex, neither in children under 8 years of age (Venham test) nor in older adolescents (CFSS).

The contradictory findings of the research may be explained by different study designs and/or data collection methods.

However, during our study, we noticed that the attitudes and behaviors of children <8 years old did not correspond to their choice of figure when they were given the Venham test, that is, we found children who arrived at the dentist very happy and whose behavior was exemplary; however, when they were given the Venham test, they selected the “anxious figure”. Based on this finding, we can hypothesize that they may be subconsciously led to feel anxious or afraid of the procedure, even if they do not outwardly show it. The contradictory findings of the research could also be explained by different study designs and/or data collection methods, and further research is needed in this area in order to clarify the results.

Regarding age, despite finding a large number of articles [17,41] that show greater anxiety in younger children, we did not find a significant relationship between the young age of the child and greater anxiety or worse behavior. However, we found a minimal correlation (*p* = 0.095) between parental anxiety and the Venham test (children > 8 years) during the first visit. This association may explain the fact that, in the case of younger children, parents are slightly more nervous than those with children who failed the CFSS (>8 years).

The pulse has no relationship with the anxiety state of children. We have found children who came to the consultation with high beats per minute (bpm) and calm behavior and a relaxed state during dental practice. This can be explained by the fact that each patient has a unique resting bpm; some are higher and others are lower. Similarly, if a child comes to the consultation after a football class or any other extracurricular activity that involves significant psychomotor movement, they may present with an accelerated pulse, which has nothing to do with a high level of anxiety. Therefore, we concluded that these data were not reliable in this regard.

As for physical manifestations, crying, movement of hands and feet, or the need for restrictions were more frequent in young patients (being more restless and curious) than in older ones; there was a relationship (*p* = 0.003) between crying and a high level of anxiety in children under 8 years of age. In addition, it was observed that the rate of patients exhibiting crying and movement of limbs increased slightly as the treatment progressed.

The most frequently performed treatments in our research were fillings, pulpotomies, pulpectomies, and tooth extractions. Thus, a statistically significant relationship was found between the treatment performed on the children and the level of anxiety of the parents; tooth extraction was the treatment that generated the most anxiety among the parents, which coincides with the results obtained in previous studies [16,20,42].

Regarding the caries index, we found a dft index of 6.73 and a DMFT index of 0.36. The explanation for these values is that, of the 101 children examined, 100 had temporary and/or mixed dentition, while only one of them had complete permanent dentition. In addition, among the patients who had mixed dentition, most did not have caries in the erupted permanent molars. For this reason, the DMFT value is significantly lower compared to the dft index.

In this regard, despite the fact that the cod index is quite high in our study, we found lower values than those reported in the study carried out by Vázquez Rodríguez et al. in 2016 [32], in which the prevalence and severity of dental caries in children aged 0 to 12 years were studied; a dft value of 7.24 and CAOD of 3.58 were found.

In contrast to our results and those of the previous study [32], Rodríguez Yáñez et al., in 2009 [43], found a low prevalence of caries in their study population, with 80% of the patients being caries-free.

According to the latest report on the global oral health situation by the WHO [44], the global prevalence of caries in temporary dentition is 43%, and it is 29% in permanent dentition; there are few variations between countries according to their income. In Spain in particular, the prevalence of caries in permanent dentition for people of both sexes aged 5 years or older is between 35.6 and 40.6%, according to the last publicated data [45].

Since this worldwide situation is alarming, the WHO’s goal is to achieve universal health coverage to remedy global deficiencies in oral health by 2030 through evidence-based preventive care, the application of fluorides, and the early diagnosis of oral diseases and their subsequent treatment in the initial stages [44].

In our study, a statistically significant relationship was found between “very high” levels of caries in DT and high anxiety, according to the Corah scale, on the part of the parents. This may lead us to think, like Rashidah et al. [46] and Sathyaprasad et al. (2024) [34], that the greater the parent’s fear of the dentist, the more they will postpone their children’s check-up visits and/or delay their children’s dental treatment, causing an increase in the children’s caries rates and a worsening of their oral health.

However, there are several articles that [4,35,47] have not found a significant relationship between maternal anxiety and oral hygiene habits in children. A very high risk of caries in DT was also found in children of parents with “no education” (50%) or only “graduate school/FP” (47.6%), compared to those who had “university education” (26.7%). This may corroborate, as has already been demonstrated by Gudipaneni et al. [48], a higher level of anxiety in parents with a low educational level and low monthly income due to ignorance and, therefore, a higher level of caries in these children.

During our study, it became clear that most mothers believed that “when we inject” the child to administer anesthesia, he would cry due to the fear this generated. However, when we explained to them that children should not know that we were going to inject them, but rather that it was a “spray”, the mothers became calmer, thus reducing their fear of the child crying. These results coincide with recent studies [33,34,49,50,51], in which it has been shown that parents who are informed about the procedures their child will undergo and about the information they should convey to their children about these procedures (explanations, demonstration videos, drawings, etc.) present a lower level of anxiety and fear of the dentist, giving rise to more cooperative and calm children during dental treatment [49].

Within the limitations of our study, we consider that the number of participants was small compared to other studies of a similar nature. Therefore, it would be interesting to repeat this study with a larger number of participants or to expand the sample.

On the other hand, another limitation of this study that we consider important in terms of the results obtained was the analysis of the degree of anxiety in the children, both the older ones (>8 years old) and the younger ones. On the one hand, we found the CFSS to be unreliable in terms of the assessment of the anxiety of these patients because it consists of general questions, and on the other hand, the Venham test was unreliable due to the subjectivity involved in the choice of images by the children. Therefore, we propose, for future research, the development of specific tests to assess dental anxiety in children.

In addition, it would be interesting to analyze how the degree of anxiety of the parents varies depending on their sex, that is, to determine, by carrying out these questionnaires or similar ones, if the degree of anxiety of fathers is greater than that of mothers, or vice versa.

## 5. Conclusions

In general, and taking into account the limitations of a study of this type, we can conclude that in the sample studied, we did not find a clear relationship between parental anxiety and the behavior of the children in the dental cabinet. However, it is true that children with higher caries rates exhibited higher levels of anxiety and worse behavior during treatment, with tooth extraction being the most unacceptable treatment.

## Figures and Tables

**Table 1:** Caries risk distribution according to the type of dentition.

DENTITION								
Category	Total N	Total %	Temporal N	Temporal %	Mixed N	Mixed %	Permanent N	Permanent %
Total	101	100.0%	46	100.0%	54	100.0%	1	100.0%
Low	1	1.0%	1	2.2%	0	0.0%	0	0.0%
Moderate	10	9.9%	10	21.7%	0	0.0%	0	0.0%
High	17	16.8%	16	34.8%	0	0.0%	1	100.0%
Very high	19	18.8%	19	41.3%	0	0.0%	0	0.0%
Low/Very low	3	3.0%	0	0.0%	3	5.6%	0	0.0%
Mod./Very low	8	7.9%	0	0.0%	8	14.8%	0	0.0%
Alto/Very low	10	9.9%	0	0.0%	10	18.5%	0	0.0%
Very high/Very low	30	29.7%	0	0.0%	30	55.6%	0	0.0%
Very high/Low	3	3.0%	0	0.0%	3	5.6%	0	0.0%

**Table 2:** Child behavior according to the Frankl scale.

FRANKL SCALE			
Category		N	%
FRANKL T1	Total	101	100.0%
	Def. positive	85	84.2%
	Lig. positive	14	13.9%
	Lig. negative	2	2.0%
FRANKL T2	Total	101	100.0%
	Def. positive	91	90.1%
	Lig. positive	10	9.9%
FRANKL T3	Total	101	100.0%
	Def. positive	85	84.2%
	Lig. positive	15	14.9%
	Lig. negative	1	1.0%

**Table 3:** Relationship between VENHAM in children and pulse/manifestations: results of Pearson correlation coefficient (r), Chi2 test, and Fisher’s exact test. * *p* value < 0.05.

		r; *p*-Value	Chi2/Fisher; *p*-Value
PULSE	T1	r= 0.06; *p* = 0.575	*p* = 0.801
	T2	r= −0.01; *p* = 0.919	*p* = 0.838
	T3	r= −0.09; *p* = 0.411	*p* = 0.819
CRYING	T1		*p* = 0.193
	T2		*p* = 1.000
	T3		*p* = 0.003 *
ARM MOV.	T1		*p* = 0.385
	T2		*p* = 0.501
	T3		*p* = 0.105
LEG MOV.	T1		*p* = 0.279
	T2		*p* = 1.000
	T3		*p* = 0.084
ORAL RESIST.	T1		*p* = 1.000
	T2		*p* = 1.000
	T3		*p* = 1.000
PHYSICAL RESIST.	T1		*p* = 1.000
	T2		*p* = 1.000
	T3		*p* = 1.000

**Table 4:** Relationship between ECOHIS of parents and treatments: results of independent samples t test and Mann–Whitney test. * *p* value < 0.05.

		t; *p*-Value	MW; *p*-Value
OBTURATION	T2	*p* = 0.207	*p* = 0.189
	T3	*p* = 0.049 *	*p* = 0.189
PULPOTOMY	T2	*p* = 0.803	*p* = 0.834
	T3	*p* = 0.181	*p* = 0.625
PULPECTOMY	T2	*p* = 0.327	*p* = 0.573
	T3	*p* = 0.050	*p* = 0.040 *
EXODONTIA	T2	*p* < 0.001 *	*p* = 0.008 *
	T3	*p* = 0.038 *	*p* = 0.024 *

**Table 5:** Relationship between CORAH of parents and treatments: results of independent samples *t* test and Mann–Whitney test. * *p* value < 0.05.

		t; *p*-Value	MW; *p*-Value
OBTURATION	T2	*p* = 0.324	*p* = 0.527
	T3	*p* = 0.536	*p* = 0.471
PULPOTOMY	T2	*p* = 0.553	*p* = 0.328
	T3	*p* = 0.222	*p* = 0.046 *
PULPECTOMY	T2	*p* = 0.147	*p* = 0.097
	T3	*p* = 0.509	*p* = 0.449
EXODONTIA	T2	*p* = 0.583	*p* = 0.665
	T3	*p* = 0.856	*p* = 0.785

**Table 6:** Relationship between STAI-Parent traits and treatments: results of independent samples *t* test and Mann–Whitney test. * *p* value < 0.05.

		t; *p*-Value	MW; *p*-Value
OBTURATION	T2	*p* = 0.010 *	*p* = 0.365
	T3	*p* = 0.513	*p* = 0.634
PULPOTOMY	T2	*p* = 0.985	*p* = 0.728
	T3	*p* = 0.763	*p* = 0.455
PULPECTOMY	T2	*p* = 0.042 *	*p* = 0.506
	T3	*p* = 0.416	*p* = 0.132
EXODONTIA	T2	*p* = 0.588	*p* = 0.813
	T3	*p* = 0.596	*p* = 0.875

**Table 7:** Relationship between cod, DMFT, risk of caries in temporary dentition, TD, and mixed DM of children and ECOHIS of parents: results of Pearson correlation coefficient (r) and Spearman (rS).

	r; *p*-Value	rS; *p*-Value
dft	r = 0.19; *p* = 0.056	--
DMFT	r = 0.12; *p* = 0.395	--
DT risk	--	rS = 0.02; *p* = 0.888
DM risk	--	rS = 0.13; *p* = 0.365

**Table 8:** Relationship between dft, DMFT, risk of caries in temporary dentition, TD, and mixed DM of children and STAI-Trait of parents: results of Pearson correlation coefficient (r) and Spearman (rS). * *p* value < 0.05.

	r; *p*-Value	rS; *p*-Value
dft	r = 0.07; *p* = 0.483	--
DMFT	r = 0.10; *p* = 0.462	--
DT risk	--	rS = −0.03; *p* = 0.865
DM risk	--	rS = 0.28; *p* = 0.040 *

## Data Availability

Data will be available by corresponding author.
